# Developing a conceptual partner selection framework for matching public–private partnerships of rural energy internet project using an integrated fuzzy AHP approach for rural revitalization in China

**DOI:** 10.1016/j.heliyon.2024.e31096

**Published:** 2024-05-10

**Authors:** Renjie Li, Mingxuan Zhang, Shi Yin, Nan Zhang, Tahir Mahmood

**Affiliations:** a School of Ethnology and Sociology, Minzu University of China, Beijing 100081, China; b College of Economics and Management, Hebei Agricultural University, Baoding, 071001, China; c College of Humanities and Social Sciences, Hebei Agricultural University, Baoding, 071000, China; d Department of Mathematics and Statistics, International Islamic University Islamabad, Pakistan

**Keywords:** Rural clean energy, Energy internet, PPP projects, Partner selection, Rural revitalization

## Abstract

The development of rural clean energy is the key to cope with the shortage of traditional energy supply in the rural revitalization strategy and improve the sustainability of rural energy supply. Under the background of digital age, the development and utilization of rural clean energy Internet has become the focus of rural economic development. The government partners of the Rural clean Energy Internet PPP project (RCEIPPPP) are the key to promoting the green and intelligent development of rural energy. In this paper, the index system of project partner selection is constructed, and the problem of government partner selection for RCEIPPPP is studied by AHP and fuzzy comprehensive evaluation. The results of this study are as follows: 1) Partners' financial ability, technical ability, management ability, performance experience, corporate reputation, cooperation ability and risk management are the influencing factors for government partner selection of rural clean energy Internet PPP projects (RCEIPPPPs); 2) Compared with other factors, financial ability, technical ability, management ability and performance experience are the four key factors that are more important in choosing partners; 3) The empirical research shows that AHP, fuzzy comprehensive evaluation and the index system constructed by this research can be applied to the practice of government partner selection for RCEIPPPPs. This study puts forward the evaluation system of government cooperation selection of energy Internet PPP projects from the theoretical level, improves the existing research methods, and makes the theoretical system in this field more complete. From the practical level, it provides scientific basis and suggestions for the government to make decisions on energy Internet PPP projects, and improves the engineering efficiency and quality of rural clean energy Internet construction. This study demonstrates the complexity of clean energy projects, the need for an integrated approach to decision-making, and the need for project managers to actively manage communication and collaboration with partners to ensure successful project implementation.

## 1 Introduction

Since the implementation of China's rural revitalization strategy, although there have been no small development and breakthroughs in rural economy, humanities and other aspects, but the rigid constraints of resources and environment are tightening, the traditional driving forces supporting farmers' income are gradually weakening, and new driving forces need to be cultivated. Clean energy can significantly reduce the dependence on traditional energy, thereby reducing greenhouse gas emissions and other environmental pollution, and comprehensively contribute to rural revitalization [1]. Therefore, the development of rural clean energy has become the key to improve the sustainability of rural energy supply, which has also been widely concerned at home and abroad. With the rapid development of China's rural economy and the acceleration of urbanization, the energy demand in rural areas is also growing. However, due to the problems of environmental pollution and resource consumption in traditional energy utilization, the development and utilization of rural clean energy has become very important, and promoting the development of rural clean energy is an effective way to improve farmers' quality of life and reduce air pollution. Rural areas are often relatively short of resources, and excessive use of traditional energy will not only lead to resource depletion, but also pose a threat to the health of farmers. However, clean energy can provide sustainable energy supply for rural areas due to its inexhaustible use [2]. According to the comparison of two national agricultural censuses, the energy consumption structure has changed to a diversified energy consumption structure that combines clean energy such as electricity and natural gas with traditional solid energy, and the proportion of rural household energy and electricity has increased by 57.8 %. The proportion of gas increased by 37.4 %, the proportion of firewood decreased by 16 %, and the proportion of coal decreased by 2.2 %. The use of clean energy met the growing energy demand in rural areas and promoted the sustainable development of rural economy [3]. In addition, the rural infrastructure construction is relatively lagging, the traditional energy supply is unstable, resulting in residential -electricity difficulties, insufficient lighting and other problems, rural clean energy has become a strong support for rural revitalization and agricultural and rural modernization of energy development outlet and important means, rural areas to promote clean energy has become the top priority to solve the energy problem.

On July 4, 2023, the National Development and Reform Commission and other jointly issued the “Guidance on the implementation of rural power grid consolidation and upgrading Project” stressed that “the construction of intelligent and open modern rural power grid, fully carrying the development and utilization of distributed renewable energy and local consumption”, so the construction of rural clean energy Internet has become the focus of national attention. The construction of rural clean energy Internet is an important direction of China's energy development at present, and it is also an inevitable choice to realize the strategy of rural revitalization. First of all, the construction of rural clean energy Internet can promote the development of rural communities and improve the quality of life of residents. By increasing the use of renewable energy and realizing the stability and intelligence of power supply, it can not only improve the energy supply situation in rural areas, but also provide more convenient, efficient and environmentally friendly services. Secondly, the construction of clean energy Internet can promote the transformation of rural energy structure, reduce dependence on traditional energy, reduce greenhouse gas emissions, improve air quality, and protect the ecological environment. The renewable energy (such as solar energy, wind energy, etc.) used in the Internet system is a green and clean form of energy that does not produce toxic waste and pollution. Relevant research results show that, from the perspective of the whole industry chain, a multi-phase, market-oriented rural energy business model is proposed, and there are certain difficulties in the development of rural energy. It is essential to fully unleash the initiative of both supply and demand. Users should actively engage in demand response efforts to enhance the alignment between supply and demand, thereby fostering the development of the entire energy supply chain. This approach will help optimize the profitability trajectory of the business model [4]. Finally, the construction of rural clean energy Internet helps to improve the stability and supply capacity of energy supply in rural areas. The use of local renewable energy resources, such as photovoltaic power plants, wind power generation, etc., can provide reliable power supply for rural areas to meet the needs of agricultural production, rural industry and residents' lives. In addition, the development of the clean energy industry chain will also drive employment growth, technological innovation and economic growth in related industries, promote the upgrading of rural industries and improve farmers' incomes. So, how to efficiently build a rural clean energy Internet? As a new form of project promotion in recent years, PPP project has become the main content of this paper.

PPP project is a public-private cooperation mode, which aims to realize the construction, operation and maintenance of public infrastructure through the resources, technology and experience of the government and private sector. With the continuous practice and evolution of the mode, the PPP mode has gradually formed the characteristics of diversified funding sources, high efficiency and flexibility, risk sharing and strong innovation ability. The importance of the rural clean Energy Internet PPP project (RCEIPPPP) is to promote the development of rural clean energy, improve the reliability of energy supply, and promote the development of rural economy and improve the quality of life of residents. On the one hand, the RCEIPPPP can promote the development and utilization of rural clean energy, and integrate various energy resources, including solar energy, wind energy, bioenergy, etc., to meet rural energy needs. Through the construction of clean energy power stations, distributed energy systems, etc., we can reduce the dependence on traditional energy sources, reduce carbon emissions, and achieve the goal of energy conservation and emission reduction [ [5]]. On the other hand, due to the lag of power supply facilities in rural areas, there is the problem of unstable power supply, which affects the normal life and production of rural residents. The RCEIPPPP can use advanced smart grid technology to achieve two-way networking of clean energy and traditional energy, form a diversified energy supply model, and improve the reliability and stability of rural energy supply. The construction of RCEIPPPPs will drive the development of related industrial chains, such as photovoltaic cells, wind power generation equipment, energy storage technology, etc., while creating job opportunities, enhance the vitality and competitiveness of the rural economy, PPP projects will become an irreplaceable necessary link in the construction of rural clean energy Internet [ [6]]. Therefore, it is a particularly critical issue to study what factors will affect the construction of PPP projects for rural clean energy Internet, and then put forward reasonable suggestions for promoting rural energy intelligence.

According to the existing literature, there are abundant researches on the selection of government partners for energy Internet and PPP projects. The construction of energy Internet can solve the problem of energy security in China from the perspectives of supply channels and import channels, and the establishment of the world energy network will help realize the optimal distribution of energy resources [ [7]]. We should not only pay attention to top-level planning and path design, but also carry out scientific analysis and evaluation on the feasibility of investment and operation of specific projects as well as the risks they face. In order to rebuild and optimize key strategic factors, dynamic capabilities must be available and play a decisive role in these factors. China's geopolitical influence and global green and low-carbon development path should also be taken into account [ [8]]. The academic community has conducted in-depth research on energy Internet from different perspectives and cases, taking the electrical and electrical appliance manufacturing industry in Shandong Province as an example, to explore the role and performance of environmental identification, organizational change, learning innovation and comprehensive coordination ability in the strategic transformation of enterprises. In essence, the strategic transformation of enterprises is to achieve the match between internal factors and external environment. Throughout the exploration process of government partner selection in PPP projects, it can be seen that Zhang et al. [ [9]] summarized the dimensions and evaluation indicators of PPP private partner selection through the method of literature frequency survey, and empowered each evaluation dimension and index with the method of entropy weight. Through the research, it was found that In the evaluation of the selection of private partners for PPP infrastructure projects, the financial capacity and business reputation of the private sector occupy a large proportion. Cong et al. [ [10]] established a collaborative partner selection and decision model of social capital alliance based on the characteristics of the cloud model that qualitative and quantitative expressions can be mutually transformed, as well as the characteristics of grey correlation method, such as high information utilization efficiency and simple data processing. The example analysis proves that the optimization decision index system and model can effectively evaluate and predict the project and relationship factors, and can reasonably determine the priority order of various alternative partners, so as to provide a decision basis for the selection of alternative partners. Li et al. [ [11]] studied three different PPP project payment methods (government payment method, user payment method and feasibility gap subsidy method), and based on this, established a mixed market PPP model including private enterprises and state-owned enterprises, and analyzed the efficiency of government-enterprise cooperation from the perspective of maximizing social welfare. Furthermore, the selection of the optimal project partner and project mode for the government is discussed, but there is a lack of specific discussion on the mechanism of rural clean energy Internet and practical research on the government's choice of cooperation objects.

Therefore, this paper focuses on the selection of government partners for RCEIPPPPs, in order to supplement the existing literature. This paper attempts to study the selection of government partners for RCEIPPPP to reveal the impact of the selection of government partners in PPP mode on rural clean energy Internet, so as to enrich the analysis perspective of PPP mode and government partner selection for energy Internet. This paper uses the literature reading method to give the government partner selection index system, and classifies the rural clean energy Internet energy enterprises, involving qualitative indicators and quantitative indicators. The entropy weight method is used to standardize the qualitative and quantitative indicators in a unified manner, and the government partner indicators of each bidder are jointly evaluated to obtain the comprehensive evaluation coefficient of each enterprise. Through the robustness test of the coefficient, the selection index of the government partner selection of the rural energy Internet PPP project is finally obtained.

The contribution of this paper is to put forward the government cooperation selection evaluation system for energy Internet PPP projects, and for the first time innovatively use AHP and fuzzy evaluation methods to select and make comprehensive decisions on PPP project partners, improve the existing research methods, and make the theoretical system in this field more complete. It also provides scientific basis and suggestions for practical decision-making of energy Internet PPP projects. In the process of combining the realistic case analysis of the rural energy engineering project, the decision-making opinions obtained by the evaluation system constructed in this paper are consistent with the decision-making results of the realistic case, and the PPP project promotes the leapfrog development of the economy and society of the county where it is located, and gets a good response, which also reflects the rigor and science of this paper to a certain extent, and also reflects a certain reference significance.

The remaining structure of this paper is as follows. Section 2 is a literature review from three aspects: energy Internet PPP project management, PPP project partner selection criteria and PPP project partner selection methods. Section 3 introduces the evaluation factors of PPP project partner selection and the partner selection system of RCEIPPPP in detail. Section 4 is the method presentation part of this paper, which introduces the five steps of PPP project partner selection and evaluation; Section 5 is the case analysis, using the real data to implement the method, and the case results are discussed; Section 6 is the conclusion of this paper.

## 2 Literature review

### 2.1 Energy internet PPP project management

The research on the energy Internet PPP project in foreign theoretical circles originates from the PPP model proposed by the British government in the 19th century. Since the further promotion of economic globalization, the PPP development of energy Internet has been highly concerned and valued by various academic circles at home and abroad, and has become a hot spot of concern for many scholars, and more research results have been achieved. These achievements can be summarized from three aspects: energy PPP project management, Internet PPP project management and Internet basic project management.

The research on energy PPP project management can be divided into two aspects: the application of energy PPP and the extension of energy PPP. PPP model is a new model of energy project financing. China introduced the PPP model for the first time in 1995. Chen and Chen [[12]] analyzed the PPP model of the energy-saving partnership development model of the US Virgin Islands and the integrated development model of the Dutch Voluntary environmental Agreement from the perspective of foreign countries, and proposed that the PPP model of energy-saving emission reduction in China needs to clarify the government's positioning, grasp technological innovation and application. The key points that need to be paid attention to when promoting the PPP mode of energy saving and emission reduction are emphasized. Bai and Tan [ [13]] introduced in detail the connotation and development process of PPP model in broad and narrow sense, and pointed out the construction and operation status of new energy electric vehicle charging facilities. PPP model is a new financing model, which aims to introduce social capital to carry out value-added services through PPP model. It is conducive to improving the construction and operation efficiency of new energy vehicle charging facilities, but there is a lack of PPP financial policy guarantee. Cao et al. [ [14]] discussed the optimization of the service mode of the integrated energy system. From the perspective of project investment and construction, they deeply analyzed the operation links and modes of the integrated energy project under different service modes, summarized and proposed the service mode suitable for the integrated energy system, and proposed the selection method of the mode. The service model evaluation system based on improved fuzzy comprehensive evaluation method is established. In the era of energy Internet, it is urgent for energy enterprises represented by power grid enterprises to adapt to the reform of the power system by transforming to comprehensive energy service providers and actively carrying out comprehensive energy services. Song et al. [ [15]] expanded the pricing method of asset securitization in PPP mode of new energy power, and conducted a case study using binary tree option pricing model for option-adjusted spread method. The study increased the financing efficiency of new energy power construction and promoted the market flow of asset securitization in PPP mode. Yang [ [16]] discusses the impact of social capital control on PPP project investment, and proposes that the higher the social capital control in China's energy PPP projects, the greater the investment in PPP projects; The longer the contract term of energy PPP projects, the less the investment of PPP projects, etc. This study provides a reference for social capital to participate in the investment decision of energy PPP projects. Wang [ [17]] combined the EMC-PPP model, took the energy-saving renovation project of an old residential area as the project, combined the energy-saving service management technology of an energy-saving company with the funds of social capital, solved the problem of difficult financing in EMC mode, and realized the complementary advantages of all participants. Based on the above, this paper proposes improvement methods for energy PPP projects: first, clarify the role of the government, relevant departments should improve the implementation system of energy PPP model; The second is to promote the expansion and innovation of the energy PPP model to promote the continuous innovation of technology.

The research on the management of Internet PPP projects can be divided into two aspects: the origin of Internet PPP projects and the expansion of Internet PPP projects under different backgrounds. There are few researches on Internet PPP projects abroad, but the research on this project in China comes from the application of PPP projects in Internet. Zeng [ [18]] analyzed the protocol structure and operation mechanism, and described its application in remote access technology. Keywords: remote access server, whose research and development is of great significance. These two studies opened up the history of China's PPP into the Internet and laid the foundation for the further development of Internet PPP projects. Ju [ [19]] analyzed the operation strategy of the “Internet + PPP” model from the perspective of SWOT model, formulated the strategic plan of the “Internet + PPP” model under the background of new urbanization, and constructed the PPP Internet platform was of great practical significance for promoting the construction of new urbanization and maintaining the steady development of China's economy. However, the analysis from this perspective is not profound enough, and some scholars have conducted in-depth exploration of “Internet + PPP” from different perspectives. Tang and Rui [ [20]] after analyzing the driving mechanism of Internet + collaborative integration, financing innovation model and appropriate evaluation method of VFM, proposed that the Internet + financing innovation model should be introduced when promoting the PPP model, which has a profound impact on the allocation of economic, cultural, environmental and social resources. Zhang and Yang [ [21]] summarized the relevant experience of Germany and the United States in applying the SPV-based PPP model to accelerate R&D breakthroughs in the field of integration and innovation, so as to compare the existing problems in China. However, the SPV model is too simple, and no effective solutions have been obtained for specific situations that are not applicable, and the foreign experience also has certain inapplicability in China. Shen et al. [ [22]] established the risk degree evaluation index system; The improved operator (ICUOWGA) is used to aggregate each index for many times, and the final comprehensive risk measurement value and the weight ranking of main factors are obtained. This method also introduces fuzzy semantic quantization operator and normalized decision matrix formula, which overcomes the shortcoming of the traditional method. Based on the above, this paper puts forward some improvement methods for Internet PPP project management: First, adopt foreign Internet PPP model according to the actual situation of our country; The second is to establish a risk evaluation index system to control risks and avoid subjective decision misjudgment.

The research on Internet infrastructure is relatively rich, mainly reflected in the analysis based on a certain perspective. ShiltonK [ [23]] argues that the participatory observations of a team developing Internet protocols show that the difficulty in defining stakeholders in infrastructure and the tension between local and global perspectives are complex value reflections, suggesting that Internet architects tend to equate the core value of interoperability with value neutrality. Ma et al. [ [24]] proposed to study the protection of Internet infrastructure against link flooding attacks from the perspective of technological economy, arguing that mitigating and defeating LFA is particularly challenging and global networks are increasingly threatened, and proposed two novel mechanisms to mitigate flooding attacks. Cooperation among autonomous systems is stimulated by incentive design and Nash bargaining. SajaniJ et al. [ [25]] established a hierarchical conceptual model to understand the preemptive strategy in PPP projects and its relationship to management issues. The study confirmed that the research results confirmed the importance of stakeholder participation in alleviating problems in PPP projects and related strategies. Lynch [ [26]] used CWNGuifi.net in Barcelona as a research object to explore how the sharing of Internet infrastructure relies on the practice of knowledge sharing, which complicates the imagination of the free flow of information without abandoning the possibility of an Internet-based public space. The research is conducive to making the Internet a digital public place and bringing a more just and equitable future for innovation in network technology. Kou and Xu [ [27]] focused their research on the practical problem of how Internet infrastructure affects carbon emission efficiency. Based on the global Malmquist-Luenberger index model of relaxation variables, static panel and panel quantile regression models were used to estimate carbon emission efficiency. The study reveals how local government intervention and market segmentation can break the bottleneck of the impact of Internet infrastructure on carbon emissions, and how governments can continuously optimize the external environment to ensure the positive impact of Internet infrastructure on carbon emissions performance. Based on the above, the improvement method proposed in this paper for the study of Internet infrastructure is to consider establishing scientific values and strengthening the interaction between Internet infrastructure and local government intervention and market segmentation.

### 2.2 PPP project partner selection criteria

In recent years, the research on PPP project partner selection criteria has been highly concerned and valued by academic circles at home and abroad, and rich research results have been achieved. These results are mainly from the focus of PPP project partner selection and research examples. Wang et al. [ [28]] used literature research and expert interviews and other methods to build an indicator system for partner selection, determined the weight of indicators with ANP method, and proposed reasonable allocation of weights according to their importance, which played a certain guiding role in the selection of partners in practice. Chen et al. [ [29]] analyzed the role of contract functions and information transparency in PPP project cooperation, and believed that the efficient selection of partners could be promoted by strengthening trust among procurement partners. Hu et al. [ [30]] identify the financial risks of PPP projects to compensate for the failure in financial risks between the government and SPV due to the lack of control over financial risks. The study found that risk analysis, identification and management to deal with uncertainties and contingencies in scope, time, cost and quality is the best choice for the financial risk management of PPP projects, and the study is conducive to further improving the financial risk of public-private partnerships and their funding sources for successful public-private partnership projects. Li et al. [ [31]] proposed that the PPP + EPC model provides an effective way to solve the investment and financing problems of large-scale public infrastructure construction and improve the construction quality of complex infrastructure projects. Taking the Yiwu Mall Tunnel project in Zhejiang Province as an example, the organizational structure, operation process and responsibilities of construction participants of the design-led PPP + EPC project were analyzed. Summarizing the experience and practice of design-led PPP + EPC mode has great reference value for the implementation of related projects. Nair and Zhang [ [32]] proposed that EUG Energy chose more partners for the key links of smart grid projects, including smart meters, unified network platform, and assisting enterprises to manage the implementation of smart grid and utility transformation. Grondys and Malinauskaite are also suggested that the enterprise ecosystem helps customers realize the full potential of their sustainability and energy management programs [ [33],[34]. Hu [ [35]] In order to better analyze the performance and improve the design of industrial alarm systems, several industrial case studies were conducted based on alarm data collected from oil sands mining plants operated by Suncor Energy Company in northern Alberta, Canada. This study shows the practicability and usefulness of advanced alarm management tools for alarm rationalization and daily alarm management. Andreas et al. [ [36]] (2016) studied the energy saving potential of the battery-assisted fleet of trolleybuses. This project was completed jointly with an industrial partner and received financial support from the Swiss Federal Energy Agency (10.13039/501100005380SFOE), thus the partnership improved the operation effect of the trolleybuses. Kazemilari et al. [ [37]] present an initial analysis of renewable energy companies listed on the stock exchange using the minimum spanning trees approach. Aba-Sah [ [38]] proposed that waste-to-energy projects require complex value chains to operate effectively. In order to identify business partners, plant operators need to establish links with organizations whose strategic objectives are consistent with their own, and conclude that supplier organizations need to find out their position in the value chain and explain the partnership based on the value of the partner organization. Raghuvanshi et al. [ [39]] argue that some countries around the world are pursuing energy security to ensure the smooth running of their economies and clean energy supply, and this study provides a multi-dimensional tool that can help build successful strategic alliances to achieve energy security. In addition, the study could facilitate different stakeholders to measure the success of any strategic alliance in the energy sector. Based on the above, this paper proposes improvement methods for the selection criteria of PPP project partners: First, multi-level partners to promote the steady progress of all major links in PPP projects; The second is to understand the value concept of partners and encourage their technological innovation to improve the cooperation compatibility of PPP projects.

### 2.3 PPP project partner selection method

The criterion of PPP project partner selection provides reference for the PPP project partner selection method, and the discussion of its selection method by senior students at home and abroad has been deepened. Xu et al. [ [40]] introduced the concept of resource confidence and proposed a cross-enterprise project partner selection model considering resource confidence, so as to minimize the risk of cross-enterprise project failure and delay caused by the uncertainty of partner resources. Within a certain range, this model can find the optimal solution in a short period of time with a high probability. He [ [41]], aiming at the problems of PPP partner selection and investment incentive contract in the medium or operation period of public project investment, established a cooperative and joint investment contract and subsidy model under existing conditions, analyzed the impact of government management efficiency on cooperative investment contract, and provided a basis for decision-making on PPP partnership investment. Song and Xu [ [42]], in order to solve the problem that the excessive satisfaction level of individual decision makers leads to the uncoordinated decision-making behaviors of individual decision makers in PPP project group decision-making, designed the scheme by reducing and correcting the satisfaction level of each decision subject, and obtained the satisfactory solution under multi-objective group decision-making by using iterative algorithms. Yan [ [43]] analyzed the implementation of PPP from the perspective of audit, emphasizing the possible problems in performance responsibility, bidding procedures, contract management, operation and implementation of partnership, so as to effectively promote the better application and development of PPP. Zhang et al. [9] considered the whole process of PPP project construction, built an evaluation index system for partner selection of infrastructure projects under the PPP model, introduced the entropy weight theory to determine the weights of each index, established a multi-level fuzzy comprehensive evaluation model, and cited examples to verify the practicability and scientificity of the model. Wu and Wu [ [44]] took the real estate projects of pension institutions as research objects, analyzed the characteristics of PPP projects of pension institutions in terms of service level, liability risk, government supervision and other aspects, as well as the advantages and disadvantages of potential partners with the government, so as to provide decision-making reference for the government to choose suitable partners. Yi [ [45]], taking the selection of private partners for PPP rail transit projects as the research object, proposed a three-stage selection mechanism including access regulation, selection procedure and negotiation through literature research and reference to two-stage selective bidding for international projects. The conclusion is objective and effective. Cong et al. [10] built a partner selection and decision model of social capital consortium based on qualitative description and quantitative representation and grey correlation method through the system integration cloud model, focusing on the partner selection and evaluation of social capital consortium under the PPP model. Tong et al. [ [46]] elaborated the formation mechanism of resource complementarity from the perspective of cooperative game, proposed a solution method based on expected return constraint selection, and constructed a project partner selection model based on resource complementarity with two-layer programming. Feng et al. [ [47]] studied the matching relationship between government and enterprises, applied grey target decision theory to quantitatively process the evaluation information of various indicators between government and enterprises in PPP projects, and finally built a public-enterprise matching model for PPP projects, which is conducive to the expansion and extension of public-enterprise partner selection methods for PPP projects. Wang et al. [ [48]] pointed out that in the early stage of PPP projects, the indicators selected by private partners would be uncertain and fuzzy, and adopted the binary semantic method to integrate and evaluate quantitative and qualitative indicators in a unified manner to obtain the functional evaluation value (comprehensive evaluation value) of each enterprise's corresponding indicators. In combination with each scheme, the partner with the highest value coefficient is selected as the most priority for the PPP project. Bisello et al. [ [49]] The urban energy district project introduces technological innovation in the energy system and brings immaterial benefits to the city, which are not properly understood and considered. This study investigates the group knowledge and group thinking on this topic through the method of the world cafe, laying a foundation for the change of end-user behavior and intangible assets. Shan and Xu [ [50]] Tax incentives play an important role in the promotion of PPP model. The tax relationship between PPP partners is an important economic lever to adjust the interest balance of partners. The impact of tax incentives on the financial balance of PPP projects is analyzed from the perspective of net present value. Zhang et al. [ [51]] analyzed the motivation and selection tendency of energy-using institutions in selecting EPC project partners, assumed the selection tendency of partners in different procurement environments, verified through successful EPC cases, and put forward appropriate approaches and relevant suggestions for energy-using institutions' partner selection. Li [ [52]] studied the measurement scale of the impact of partner selection indicators on performance, and built the impact model of selection indicators, which provided certain references for scientific allocation of PPP partner selection indicator weights and improvement of PPP project performance. However, it only included static analysis and lacked certain path quantitative reasoning. Through descriptive statistical analysis method, Yin and Zhao ^[53]^ the driving mechanism of rural new energy connection is deeply discussed to provide guidance for optimizing the construction of rural new energy system. Yin and Zhang ^[54]^ analyzed the pressure-state-response (PSR) model of digital technology to promote green innovation in manufacturing industry, and built the measurement system of digital green innovation in manufacturing industry according to the PSR framework, which promoted the green intelligent development of manufacturing industry. Yu and Yin ^[55^,^56]^explores and clarifies the entanglement mechanism between new energy enterprises and village collective, constructs the quantum evolutionary game model of new energy enterprises and venture capital enterprises, and introduces a set of quantum strategies for both sides, clarifies the entanglement mechanism between new energy enterprises and village collective, and promotes the smooth implementation of regional ecological environment planning and planning process. Li et al. [11] took cooperation efficiency as the starting point, introduced three types of PPP payment models, including government payment, user payment and feasibility gap subsidy, built a mixed-market PPP model, analyzed the efficiency of government-enterprise cooperation from the perspective of social welfare maximization, and discussed the selection of optimal government PPP project partners. Based on this, this paper proposes improving methods for PPP project partner selection: first, strengthen the practicability of PPP project partner selection model; The second is to enrich the dynamic model research of PPP project partner selection method.

## 3 Analysis of energy internet PPP project partner selection factors

### 3.1 Energy internet PPP project partner role analysis

Partnership is the highest level of cooperation between people or between companies, it refers to the full communication on the basis of mutual trust, challenging improvement plans for the common goal, and strict criteria to measure cooperation performance and continuous improvement. In the whole life cycle of construction and operation, the rural clean energy Internet project using the PPP model involves multiple stages including financing, design and construction. At each stage, private partners from different sectors will participate in the project operation and share risks and benefits. Therefore, the roles of private sector partners should include project financing roles, technical roles, management roles, cooperation roles, and risk management roles.

(1) Project financing role. The promotion of the rural clean energy Internet PPP model can effectively alleviate the financial pressure caused by the lack of national funds while reducing the environmental pollution and resource consumption caused by the traditional energy model. Among them, the introduction of the PPP model is mainly completed by energy Internet enterprises, so enterprises should not only have a good asset situation and capital liability ratio, but also develop a clean energy Internet fund use plan, and ensure the rationality of the financing plan.
(2) Technical role of the project. In the engineering construction of the energy Internet, the technical role plays a core role, and the embodiment of the technical role also includes many aspects, including the rural human resources of the enterprise, the construction management, maintenance and operation of the clean energy Internet, core technologies, and facility transfer schemes. Internet construction projects in rural areas are facing more complex situations, so in the process of introducing the PPP model, attention should be paid to partners' control and application of technology, and technical solutions should be formulated for possible problems.
(3) Project management roles. The infrastructure construction of clean energy Internet in rural areas has a huge investment, a long construction period, and a large number of participants. Project managers must conduct comprehensive management of all aspects of the project from investment decision, design, construction to operation. To this end, enterprises should be able to fully cooperate and communicate in the implementation process of PPP projects, and ensure the rationality of the organizational structure of rural PPP projects, the perfection of the clean energy Internet management system and the ability to operate and maintain the clean energy Internet, so as to achieve the expected purpose of the project and undertake the construction task of the project.
(4) Project risk management roles. In the process of construction of rural clean energy Internet project, the risk in the construction process of Internet project is an inevitable objective phenomenon, and reasonable risk allocation is of great significance for the achievement of project objectives and the improvement of project performance. The private partnership introduced by the government should have the Internet risk management system process specifications to avoid the loss of project cost caused by excessive project cost.

### 3.2 Energy internet PPP project partner selection evaluation dimension analysis

**Fig. 1:**
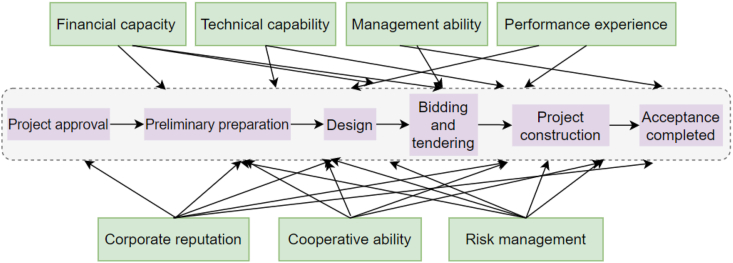
Energy Internet PPP project partner selection evaluation system division.

Source: This figure is drawn by the authors

PPP project operation process usually includes project initiation, preliminary preparation, design, bidding and tendering, project construction, acceptance and completion of six stages. Partners selected for RCEIPPPPs must have comprehensive capabilities and qualities. According to the literature review, domestic and foreign scholars have different views on the dimensions of partner selection in the PPP model. Wang et al. [27] divided the evaluation system into a preliminary evaluation stage and a detailed evaluation stage. In the preliminary evaluation stage, the partners of operational infrastructure PPP projects were evaluated from four dimensions, including qualification, financing and performance, and in the detailed evaluation stage, the partners were evaluated from seven dimensions, including finance, service, experience and reputation. Cao et al. [14] proposed two index systems, quantitative and qualitative, focusing on the detailed division of project risks from eight dimensions, such as political risk, financing risk, legal risk and force majeure risk. Wang et al. [48] constructed a private partner evaluation system from five aspects, including finance, technology, management, similar experience, and corporate reputation, and selected partners through a model based on binary semantics and value engineering. This paper uses a multi-level fuzzy comprehensive evaluation model, classifies the existing literature on the selection of contractors and concessioners in PPP projects, and identifies the dimensions of evaluation indicators. Through statistics, it is found that most scholars' views focus on finance, reputation, construction and operation capacity, risk management and control, contract management and other aspects, which can provide reference for the evaluation of PPP project partners. From the perspective of PPP project operation process and the role of rural clean energy Internet partners, this paper divides the selection and evaluation of partners into seven dimensions: financial capability, technical capability, management capability, performance experience, corporate reputation, cooperation capability and risk management. The corresponding relationship of each dimension is shown in Fig. 1.

In the above figure, corporate reputation and risk management run through the construction process of the entire RCEIPPPP. Good business reputation can be used as an intangible resource for private partners in the construction process of clean energy Internet in rural areas. To some extent, it can effectively predict the Internet construction behavior of partners. This is very important for the decision of the project, and the risk management ability is reflected in every key link in the whole process. In the process of building clean energy Internet facilities, financing and fund management require the financing capacity of partners; Bidding and project construction require technical ability and management ability to ensure the smooth progress of project construction; The implementation of large-scale PPP projects inevitably requires strong cooperation and risk management capabilities. The specific evaluation system and indicators included are shown in Table 1 below:

(1) Financial ability. This is a key factor to measure the strength of rural energy Internet PPP project partners, if the rural clean energy Internet project capital management ability is poor, can not carry out reasonable planning of funds, can not carry out effective financing of funds, resulting in a funding gap, which will have a great impact on the construction of Internet facilities. Therefore, the financial capacity of the enterprise includes: the rationality of the PPP project financing plan, the assets of energy Internet enterprises, the asset-liability ratio of energy Internet enterprises, the price competitiveness of rural PPP partners, the solvency of rural PPP cooperation Huoan, and the use of clean energy Internet funds.
(2) Technical ability. Internet infrastructure construction investment is huge, the project is complex, the whole process of construction and operation management requirements are difficult, partners make full use of their technical advantages, to provide customers with high-quality public goods or services. Specific indicators specifically include the following: rural human resources, clean energy Internet construction management plan, clean energy Internet maintenance and operation plan, clean energy Internet core technology resources and clean energy Internet facility transfer plan.
(3) Management ability. In the construction process of clean energy Internet infrastructure in rural areas, the project manager must conduct comprehensive management of all aspects of the project from investment decision, design, construction to operation, so as to achieve the expected purpose of the project and undertake the construction task of the project. Specific indicators include the following: rural PPP project cooperation and communication ability, the rationality of the organizational structure of rural PPP projects, the perfection of the clean energy Internet management system, and the clean energy Internet maintenance and management ability.
(4) Performance experience. It is of great significance to select the right partner and focus on rural clean energy Internet infrastructure with the advantages brought by its experience in PPP project management and energy Internet facility construction. Specific experience includes: clean energy operation experience, energy Internet construction experience, rural PPP project financing experience and rural PPP project owner satisfaction.
(5) Corporate reputation. In PPP projects, the link between the public and private sectors is not only the contracts signed by the two parties, but more importantly, the corporate credibility owned by the private sector. Therefore, in PPP projects, choosing the private sector with a good reputation as a partner can reduce transaction costs, improve construction efficiency and improve project performance. The qualification of the enterprise can reflect the operation ability of the partner from one side; Enterprise credit rating, in the past project, PPP project contract performance rate, breach of contract litigation record, enterprise energy Internet qualification level and other quality and safety records that affect the project period should be the object of key review.
(6) Cooperation ability. Clean energy Internet infrastructure PPP projects require a higher level of collaboration capacity than ordinary public-private partnerships. Among them, the consistency of the strategic objectives of the PPP project, the experience of rural communication and coordination of the partners, the satisfaction of farmers in the whole cycle of the clean energy Internet, and the strategic compatibility of the whole life cycle of the clean energy Internet are the main characteristics.
(7) Risk management. PPP projects have the characteristics of complex financing structure, huge investment scale, numerous participants, and complex relationship coordination. There are many uncertainties in the implementation and operation of rural clean energy Internet projects. The risk allocation of project parties is an important reason for the success or failure of PPP projects. Be able to effectively identify risks in the project and coordinate the relationship between project parties during the project implementation process. Among them, the financial resilience of PPP partners, the rationality of the Internet risk-sharing plan and the process specification of the Internet risk management system are all important indicators to measure the risk management level of private partners.

**Table 1:** RCEIPPPP partner selection system.

Goal	System	Serial number	Index
RCEIPPPP partner selection system	Financial capability *C*_*1*_	1	Rationality of PPP project financing scheme *C*_*11*_
		2	Energy Internet enterprise assets *C*_*12*_
		3	Energy Internet enterprise asset-liability ratio *C*_*13*_
		4	Price competitiveness of rural PPP partners *C*_*14*_
		5	Solvency of rural PPP partners *C*_*15*_
		6	Clean energy Internet Fund use plan *C*_*16*_
	Technical capability *C*_*2*_	7	Situation of rural human resources *C*_*21*_
		8	Clean energy Internet construction management plan *C*_*22*_
		9	Clean energy Internet maintenance and operation plan *C*_*23*_
		10	Clean energy Internet core technology resources *C*_*24*_
		11	Clean Energy Internet Infrastructure Transfer Program *C*_*25*_
	Management capability *C*_*3*_	12	Rural PPP project cooperation and communication ability *C*_*31*_
		13	Reasonable organizational structure of rural PPP projects *C*_*32*_
		14	Clean energy Internet management system perfection *C*_*33*_
		15	Clean energy Internet operation and maintenance management capability *C*_*34*_
	Performance experience *C*_*4*_	16	Clean energy operation Experience *C*_*41*_
		17	Energy Internet construction experience *C*_*42*_
		18	Rural PPP project financing experience *C*_*43*_
		19	Satisfaction of owners of rural PPP projects *C*_*44*_
	Corporate reputation *C*_*5*_	20	Corporate credit rating *C*_*51*_
		21	PPP project contract performance rate *C*_*52*_
		22	PPP project default litigation record *C*_*53*_
		23	Enterprise energy Internet qualification level *C*_*54*_
	Cooperation capability *C*_*6*_	24	Alignment of strategic objectives for PPP projects *C*_*61*_
		25	Partner rural communication and coordination experience *C*_*62*_
		26	Farmer satisfaction in the whole cycle of clean energy Internet *C*_*63*_
		27	Clean energy Internet full life cycle strategic compatibility *C*_*64*_
	Risk management *C*_*7*_	28	Financial resilience of PPP partners *C*_*71*_
		29	Internet construction Risk Sharing Solution Rationality *C*_*72*_
		30	Internet risk management system process specification *C*_*73*_

## 4 Methodology

### 4.1 Energy internet evaluation index system construction

This paper adopts the literature frequency statistics method, and integrates the relevant opinions of middle and senior management personnel, technical personnel, supervision engineers of enterprises who have participated in large-scale PPP infrastructure projects, as well as public sector personnel with experience in PPP infrastructure projects, so as to comprehensively consider the comprehensiveness and scientificity of evaluation indicators. Finally, the evaluation index system of partner selection for infrastructure projects under the PPP model is constructed, and the specific contents are shown in Table 1 above.

### 4.2 Energy internet evaluation method

Since most of the index factors are fuzzy, it is difficult to describe them quantitatively, so as to provide decision-making basis for the partner selection of PPP projects. On this basis, a method based on multi-layer fuzzy comprehensive evaluation is proposed, and the effectiveness of the method is verified by an example:

(1) Set of comments to determine the evaluation object

When determining the set of comments of the evaluation object, it can be divided into certain levels. Here, from the perspective of “expert scoring”, the evaluation level is divided into five levels of “10 points”, “8 points”, “6 points”, “4 points” and “2 points”, and when choosing partners for RCEIPPPP, the comments set is expressed as *V={10 points, 8 points, 6 points, 4 points, 2 points}*, The evaluation connotation of the review set is *V={benchmark level, lean level, standard level, growth level, initial level}*.

(2) Determine the membership of each indicator *u*_*i*_ to the *r*_*ij*_ of the comment in *V.*

In this paper, the membership *r*_*ij*_ was determined by the evaluation committee scoring method. If there are *n* people in the evaluation committee, then for a certain cooperative enterprise, the membership degree of an indicator in the index layer belonging to a comment in *V* is expressed as:

Since the 30 indicators in C are divided into seven categories according to the seven criteria of financial ability *U*_*1*_,technical ability *U*_*2*_, management ability *U*_*3*_, performance experience *U*_*4*_, corporate reputation *U*_*5*_, cooperation ability *U*_*6*_ and risk management *U*_*7*_, the evaluation matrix is constructed by taking the elements in each category as a whole. For example, in *U*_*1*_ (financial capacity), “Rationality of PPP project financing plan”, “Asset situation of energy Internet enterprises”, “asset-liability ratio of energy Internet enterprises”, “Price competitiveness of rural PPP partners”, “solvency of rural PPP partners”, and “clean energy Internet fund use Plan” are the five levels in the comments set *V*. The above definition gives the 6 × 5 matrix *R*_*1*_, and the same is true for *R*_*2*_*, R*_*3*_*, R*_*4*_*, R*_*5*_*, R*_*6*_*, R*_*7.*_ The results are equations (1), (2), (3).

(1) $$R_{1}=\left[\begin{array}{ccccc} r_{11} & r_{12} & r_{13} & r_{14} & r_{15}\\ r_{21} & r_{22} & r_{23} & r_{24} & r_{25}\\ r_{31} & r_{32} & r_{33} & r_{34} & r_{35}\\ r_{41} & r_{42} & r_{43} & r_{44} & r_{45}\\ r_{51} & r_{52} & r_{53} & r_{54} & r_{55}\\ r_{61} & r_{62} & r_{63} & r_{64} & r_{66} \end{array}\right]$$

(2) $$R_{2}=\left[\begin{array}{ccccc} r_{11}^{\prime } & r_{12}^{\prime } & r_{13}^{\prime } & r_{14}^{\prime } & r_{15}^{\prime }\\ r_{21}^{\prime } & r_{22}^{\prime } & r_{23}^{\prime } & r_{24}^{\prime } & r_{25}^{\prime }\\ r_{31}^{\prime } & r_{32}^{\prime } & r_{33}^{\prime } & r_{34}^{\prime } & r_{35}^{\prime }\\ r_{41}^{\prime } & r_{42}^{\prime } & r_{43}^{\prime } & r_{44}^{\prime } & r_{45}^{\prime }\\ r_{51}^{\prime } & r_{52}^{\prime } & r_{53}^{\prime } & r_{54}^{\prime } & r_{55}^{\prime } \end{array}\right]$$

(3) $$R_{7}=\left[\begin{array}{ccccc} r_{11}^{⁗} & r_{12}^{⁗} & r_{13}^{⁗} & r_{14}^{⁗} & r_{15}^{⁗}\\ r_{21}^{⁗} & r_{22}^{⁗} & r_{23}^{⁗} & r_{24}^{⁗} & r_{25}^{⁗}\\ r_{31}^{⁗} & r_{32}^{⁗} & r_{33}^{⁗} & r_{34}^{⁗} & r_{35}^{⁗} \end{array}\right]$$

(3) Fuzzy analytic Hierarchy Process to determine the weight

In the comprehensive evaluation system given in Table 1, the importance of the seven criteria and 30 indicators in the comprehensive evaluation is different. If the status is important, greater weight should be given; On the contrary, less weight should be given.

The basic principle of fuzzy analytic hierarchy process starts from the hierarchical structure of the comprehensive evaluation system, applies the knowledge, wisdom, information and values of experts to compare and contrast the indicators in the same layer or the same domain, and constructs the judgment matrix *D=(d*_*ij*_*)*_*m×n*_ according to the 1–9 judgment scale and meaning. Then, the organizer calculates the largest eigenroot *λ*_max_ of the comparison judgment matrix *D*, and has *λ*_max_ solve the eigenequation: *D*_*x*_*=λ*_*max*_*x* to obtain the eigenvector *x=(x*_*1*_*, x*_*2*_*, …, x*_*n*_*)*^*T*^ corresponding to *λ*_*max*_. Finally, the normalization process is equation (4).

(4) $$W_{A}=\left(\frac{x_{1}}{\sum_{i=1}^{n}x_{i}},\frac{x_{2}}{\sum_{i=1}^{n}x_{i}},\cdot \cdot \cdot ,\frac{x_{n}}{\sum_{i=1}^{n}x_{i}}\right)$$

Before determining the final weight vector, the consistency test of the judgment matrix *D* is needed. The weight vectors determined by any of the methods are equations (5), (6), (7), (8).

(5) $$W_{Α}=\left(\alpha_{1},\alpha_{2},\cdots ,\alpha_{7}\right)$$

(6) $$W_{C1}=\left(\alpha_{11},\alpha_{12},\alpha_{13},\alpha_{14},\alpha_{15},\alpha_{16}\right)$$

(7) $$W_{C2}=\left(\alpha_{21},\alpha_{22},\alpha_{23},\alpha_{24},\alpha_{25}\right)$$

(8) $$\cdots W_{C7}=\left(\alpha_{71},\alpha_{72},\alpha_{73}\right)$$

Where A represents the weight vector of the three criteria: financial ability *U*_*1*_, technical ability *U*_*2*_, management ability *U*_*3*_, performance experience *U*_*4*_, corporate reputation *U*_*5*_, cooperation ability *U*_*6*_, risk management *U*_*7*_; *A*_*i*_ represents the weight vector of each indicator in each criterion *U*_*i*_ (*i = 1,2,3*).

(4) Comprehensive evaluation of PPP project partners is equation (9).

(9) $$B=W_{A}ΟR_{A}=\left(a_{1},a_{2},...,a_{7}\right)Ο\left(\begin{array}{c} A_{1}ΟR_{C1}\\ A_{2}ΟR_{C2}\\ \begin{array}{c} ...\\ A_{7}ΟR_{C7} \end{array} \end{array}\right)=\left(b_{1},b_{2},b_{3},b_{4},b_{5}\right)$$

Where “O” takes the operator: ● defined as; ⨁ is defined as.

The normalization results are equation (10).

(10) $$B=\left(\frac{b_{1}}{\sum_{i=1}^{5}b_{i}}，\frac{b_{2}}{\sum_{i=1}^{5}b_{i}}，\cdot \cdot \cdot ，\frac{b_{5}}{\sum_{i=1}^{5}b_{i}}\right)\overset{\Delta }{=}\left(C_{1},C_{2},\cdot \cdot \cdot ，C_{5}\right)$$

The results show that *C*_*1*_% of the judges think that 10 points can be scored for the RCEIPPPP partner selection, *C*_*2*_% of people think that 8 points can be scored, *C*_*3*_% of people think that 6 points can be scored, *C*_*4*_% of people think that 4 points can be scored, *C*_*5*_% of people think that 2 points.

Further, 2 points, 4 points, 6 points, 8 points, 10 points as five grades. Let *Y=*(2,4,6,8,10)^*T*^, so the comprehensive evaluation of the PPP project partners is divided into: *Z=BOY*, and “O” takes the operator M(∙,+) to get equation (11).

(11) Z=2C1+4C2+6C+38C4+10C5

(5) Description of fuzzy multi-attribute decision theory

Fuzzy multi-attribute decision theory is developed on the basis of classical multi-attribute decision theory, and it can be expressed as: Given *A* scheme set *A={A*_*1*_*,A*_*2*_*, …, A*_*m*_*}* and the corresponding set of attributes (also known as the index set) *C=(C*_*1*_*,C*_*2*_*, …, C*_*n*_*)* for each scheme, and given the set of weights *w=(w*_*1*_*,w*_*2*_*, …, w*_*n*_*)* of relative importance for each attribute. The known attribute index, weight size and data structure are represented as fuzzy subsets or fuzzy numbers in the decision space, and the fuzzy index value matrix is obtained, denoted as *F=(f*_*ij*_*)*_*m×n*_. Then the generalized fuzzy synthesis operator is used to transform the fuzzy weight vector w and the fuzzy index value matrix *F*, and the fuzzy decision matrix *D: D=wⒽF* is obtained. The elements in *D* are sorted by the fuzzy discount medium decision method, so as to select the optimal scheme in *A*_*i*_*(i=1,2, …, m)*.

## 5 Case analysis

This paper takes an Internet PPP project as an example, the bidding announcement is publicly released by the media after the approval of the relevant government departments, and finally the three consortia of *A*, *B* and *C* submit the plan in accordance with the requirements. This data was obtained by expert survey, which to a certain extent represents the opinions of authoritative institutions on the selection of private sector partners for infrastructure projects under the PPP model.

### 5.1 Gets the energy internet membership matrix

According to the RCEIPPPP partner selection system established in Table 1, the three private enterprises *A, B* and C are scored through the method of expert scoring. The scoring rules are divided into five levels, which are “10 points”, “8 points”, “6 points”, “4 points” and “2 points”. The set of comments is expressed as *V={10 points, 8 points, 6 points, 4 points, 2 points}*, and the evaluation connotation is *V={initial level, growth level, standard level, lean level, benchmarking level}*. The membership matrix is constructed with enterprise *A* as the operation case in Table 2.

**Table 2:** Statistical table of expert scores in Enterprise *A*.

Index	2 points	4 points	6 points	8 points	10 points
***C***_*11*_	1	1	3	2	2
***C***_*12*_	0	2	1	4	2
***C***_*13*_	1	1	3	2	2
***C***_*14*_	2	1	3	2	1
***C***_*15*_	1	1	3	3	1
***C***_*16*_	0	3	3	3	0
***C***_*21*_	1	1	3	2	2
***C***_*22*_	0	2	1	4	2
***C***_*23*_	1	1	3	3	1
***C***_*24*_	0	1	4	2	2
***C***_*25*_	0	3	3	3	0
***C***_*31*_	1	2	1	4	1
***C***_*32*_	2	1	3	2	1
***C***_*33*_	0	1	4	2	2
***C***_*34*_	0	3	3	3	0
***C***_*41*_	1	2	1	4	1
***C***_*42*_	1	2	2	3	1
***C***_*43*_	1	1	3	3	1
***C***_*44*_	1	1	3	2	2
***C***_*51*_	0	3	3	3	0
***C***_*52*_	1	2	2	3	1
***C***_*53*_	2	1	3	2	1
***C***_*54*_	1	1	3	2	2
***C***_*61*_	0	2	1	4	2
***C***_*62*_	2	1	3	2	1
***C***_*63*_	1	2	1	4	1
***C***_*64*_	2	1	3	2	1
***C***_*71*_	1	1	3	3	1
***C***_*72*_	0	3	3	3	0
***C***_*73*_	1	1	3	2	2

According to the proportion of experts for each indicator in Table 2, where 1 is 0.11 experts, the fuzzy evaluation matrix is constructed.

*R*_*C11*_*=(*0.11, 0.11, 0.33, 0.22, 0.22*)*

*R*_*C12*_*=(*0.00, 0.22, 0.11, 0.44, 0.22*)*

*R*_*C13*_*=(*0.11, 0.11, 0.33, 0.22, 0.22*)*

*R*_*C14*_*=(*0.22, 0.11, 0.33, 0.22, 0.11*)*

*R*_*C15*_*=(*0.11, 0.11, 0.33, 0.33, 0.11*)*

*R*_*C16*_*=(*0.00, 0.33, 0.33, 0.33, 0.00*)*

Membership matrix of *R*_*C1*_ is composed of its corresponding matrices *R*_*C11*_*, R*_*C12*_*, R*_*C13*_*, R*_*C14*_*, R*_*C15*_ and R_C16_. Similarly, membership matrix R_C2_, membership matrix R_C3_, membership matrix R_C4_, membership matrix R_C5_, membership matrix R_C6_ and membership matrix R_C7_ can be obtained, and fuzzy operations can be performed on them. The results are equation (12).

(12) $$R_{C1}=\left(\begin{array}{ccccc} 0.11 & 0.11 & 0.33 & 0.22 & 0.22\\ 0 & 0.22 & 0.11 & 0.44 & 0.22\\ 0.11 & 0.11 & 0.33 & 0.22 & 0.22\\ 0.22 & 0.11 & 0.33 & 0.22 & 0.11\\ 0.11 & 0.11 & 0.33 & 0.33 & 0.11\\ 0 & 0.33 & 0.33 & 0.33 & 0 \end{array}\right)$$

### 5.2 AHP index weight determination

In this paper, the government partners of RCEIPPPP choose the weight of the evaluation index system, through the method of expert evaluation, with the help of the knowledge and experience of relevant experts, the pairwise comparison of various indicators of the system, the *1–9* scale method is used to score, multiple judgment matrices are constructed, and the weight of each system index is calculated through verification and adjustment. The judgment matrix is in Table 3, Table 4, Table 5, Table 6, Table 7, Table 8, Table 9, Table 10.

**Table 3:** *A-C* judgment matrices, weights and consistency tests.

*A*	*C*_*1*_	*C*_*2*_	*C*_*3*_	*C*_*4*_	*C*_*5*_	*C*_*6*_	*C*_*7*_	*w*_*i*_	Consistency check
***C***_*1*_	1	2	3	3	4	4	5	0.3343	CR = 0.0203; *λ*_max_ = 7.1658
***C***_*2*_	0.5	1	2	2	3	3	3	0.2091	
***C***_*3*_	0.33	0.5	1	2	2	3	2	0.1442	
***C***_*4*_	0.33	0.5	0.5	1	2	2	2	0.1116	
***C***_*5*_	0.25	0.33	0.5	0.5	1	2	2	0.0829	
***C***_*6*_	0.25	0.33	0.33	0.5	0.5	1	1	0.0582	
***C***_*7*_	0.2	0.33	0.5	0.5	0.5	1	1	0.0597

**Table 4:** *C*_*1*_*–C*_*1i*_ judgment matrices, weights and consistency tests.

*C*_*1*_	*C*_*11*_	*C*_*12*_	*C*_*13*_	*C*_*14*_	*C*_*15*_	*C*_*16*_	*w*_*i*_	Consistency check
***C***_*11*_	1	0.25	0.33	0.5	2	2	0.1060	CR = 0.0274; *λ*_max_ = 6.1724
***C***_*12*_	4	1	1	2	3	4	0.3058	
***C***_*13*_	3	1	1	2	2	3	0.2598	
***C***_*14*_	2	0.5	0.5	1	2	3	0.1716	
***C***_*15*_	0.5	0.33	0.5	0.5	1	2	0.0944	
***C***_*16*_	0.5	0.25	0.33	0.33	0.5	1	0.0624

**Table 5:** *C*_*2*_*–C*_*2i*_ judgment matrices, weights and consistency tests.

*C*_*2*_	*C*_*21*_	*C*_*22*_	*C*_*23*_	*C*_*24*_	*C*_*25*_	*w*_*i*_	Consistency check
***C***_*21*_	1	3	3	1	3	0.3331	CR = 0.0652; *λ*_max_ = 5.2923
***C***_*22*_	0.33	1	0.5	0.33	0.25	0.0733	
***C***_*23*_	0.33	2	1	0.5	2	0.1589	
***C***_*24*_	1	3	2	1	3	0.3072	
***C***_*25*_	0.33	4	0.5	0.33	1	0.1276

**Table 6:** *C*_*3*_*–C*_*3i*_ judgment matrices, weights and consistency tests.

*C*_*3*_	*C*_*31*_	*C*_*32*_	*C*_*33*_	*C*_*34*_	*w*_*i*_	Consistency check
***C***_*31*_	1	0.5	0.5	2	0.1899	CR = 0.0265; *λ*_max_ = 4.0709
***C***_*32*_	2	1	2	3	0.4203	
***C***_*33*_	2	0.5	1	2	0.2685	
***C***_*34*_	0.5	0.33	0.5	1	0.1276

**Table 7:** *C*_*4*_*–C*_*4i*_ judgment matrices, weights and consistency tests.

*C*_*4*_	*C*_*41*_	*C*_*42*_	*C*_*43*_	*C*_*44*_	*w*_*i*_	Consistency check
***C***_*41*_	1	2	0.25	0.33	0.1297	CR = 0.0304; *λ*_max_ = 4.0812
***C***_*42*_	0.5	1	0.25	0.33	0.0917	
***C***_*43*_	4	4	1	2	0.4829	
***C***_*44*_	3	3	0.5	1	0.2957

**Table 8:** *C*_*5*_*–C*_*5i*_ judgment matrices, weights and consistency tests.

*C*_*5*_	*C*_*51*_	*C*_*52*_	*C*_*53*_	*C*_*54*_	*w*_*i*_	Consistency check
***C***_*51*_	1	0.33	0.5	2	0.1671	CR = 0.0265; *λ*_max_ = 4.0709
***C***_*52*_	3	1	2	3	0.4531	
***C***_*53*_	2	0.5	1	2	0.2616	
***C***_*54*_	0.5	0.33	0.5	1	0.1182

**Table 9:** *C*_*6*_*–C*_*6i*_ Judgment matrices, weights and consistency tests.

*C*_*6*_	*C*_*61*_	*C*_*62*_	*C*_*63*_	*C*_*64*_	*w*_*i*_	Consistency check
***C***_*61*_	1	4	2	2	0.434	CR = 0.0171; *λ*_max_ = 4.0457
***C***_*62*_	0.25	1	0.33	0.5	0.098	
***C***_*63*_	0.5	3	1	2	0.2856	
***C***_*64*_	0.5	2	0.5	1	0.1825

**Table 10:** C_7_–C_7i_ Judgment matrices, weights and consistency tests.

*C*_*7*_	*C*_*71*_	*C*_*72*_	*C*_*73*_	*w*_*i*_	Consistency check
***C***_*71*_	1	3	2	0.5499	CR = 0.0176; *λ*_max_ = 3.0183
***C***_*72*_	0.33	1	1	0.2098	
***C***_*73*_	0.5	1	1	0.2402	

Note: The above tables are calculated according to AHP index.

Therefore, the weight vector of each indicator layer is shown as follows:

*W*_*A*_*=(wc*_*1*_*,wc*_*2*_*,wc*_*3*_*,wc*_*4*_*,wc*_*5*_*,wc*_*6*_*,wc*_*7*_*)=(*0.3343, 0.2091, 0.1142, 0.1116, 0.0829, 0.0582, 0.0597*)*

*W*_*C1*_*=(*0.106, 0.3058, 0.2598, 0.1716, 0.0944, 0.0624*)*

*W*_*C2*_*=(*0.3331, 0.0733, 0.01589, 0.3072, 0.1276*)*

*W*_*C3*_*=(*0.1899, 0.4203, 0.2685, 0.1276*)*

*W*_*C4*_*=(*0.1297, 0.0917, 0.4829, 0.2957*)*

*W*_*C5*_*=(*0.1671, 0.4531, 0.2616, 0.1182*)*

*W*_*C6*_*=(*0.434, 0.098, 0.2856, 0.1825*)*

*W*_*C7*_*=(*0.5499, 0.2098, 0.2402*)*

### 5.3 Fuzzy comprehensive evaluation operation

Membership matrix *R*_*C1*_ is composed of its corresponding submatrices *R*_*C11*_, *R*_*C12*_, *R*_*C13*_, *R*_*C14*_, *R*_*C15*_ *and R*_*C16*_, and the results are equation (13).

(13) $$R_{C1}=\left(\begin{array}{ccccc} 0.11 & 0.11 & 0.33 & 0.22 & 0.22\\ 0 & 0.22 & 0.11 & 0.44 & 0.22\\ 0.11 & 0.11 & 0.33 & 0.22 & 0.22\\ 0.22 & 0.11 & 0.33 & 0.22 & 0.11\\ 0.11 & 0.11 & 0.33 & 0.33 & 0.11\\ 0 & 0.33 & 0.33 & 0.33 & 0 \end{array}\right)$$

Membership matrices *R*_*C2*_*, R*_*C3*_*, R*_*C4*_*, R*_*C5*_*, R*_*C6*_ and *R*_*C7*_ can be obtained in the same way. Fuzzy operation is performed on the membership matrix, and the results are shown as follows:

*B*_*C1*_*]W*_*C1*_*•R*_*C1*_*=(*0.0893, 0.159, 0.2654, 0.3076, 0.1788*)*

*B*_*C2*_*]W*_*C2*_*•R*_*C2*_*=(*0.0388, 0.1317, 0.3035, 0.2227, 0.1603*)*

*B*_*C3*_*]W*_*C3*_*•R*_*C3*_*=(*0.1145, 0.1613, 0.3231, 0.28, 0.1275*)*

*B*_*C4*_*]W*_*C4*_*•R*_*C4*_*=(*0.1111, 0.1357, 0.2943, 0.3149, 0.144*)*

*B*_*C5*_*]W*_*C5*_*•R*_*C5*_*=(*0.1216, 0.1986, 0.283, 0.2911, 0.1057*)*

*B*_*C6*_*]W*_*C6*_*•R*_*C6*_*=(*0.0941, 0.1911, 0.1735, 0.3822, 0.1593*)*

*B*_*C7*_*]W*_*C7*_*•R*_*C7*_*=(*0.0878, 0.1577, 0.3333, 0.3066, 0.1145*)*

*R*_*A*_ is composed of its corresponding submatrix *B*_*C1*_*, B*_*C2*_*, B*_*C3*_*, B*_*C4*_*, B*_*C5*_*, B*_*C6*_ and *B*_*C7*_, which can be obtained by fuzzy calculation formula:

*B A = W*_*A*_*• R*_*A*_*=(0.0910, 0.1594, 0.2950, 0.2996, 0.1599),* the fuzzy matrix *B A* and the comments set *V={2,4,6,8,10}* by fuzzy calculation, we can get the comprehensive score of A project is *6.59,* and the project level of *A* enterprise is “lean level”.

Expert scoring method can know the expert scoring situation of enterprise *B* and *C* projects, and the results are shown in Table 11 and Table 12.

**Table 11:** B enterprise experts score statistics table.

Index	2 points	4 points	6 points	8 points	10 points
***C***_*11*_	1	1	3	3	1
***C***_*12*_	0	1	4	2	2
***C***_*13*_	2	1	3	2	1
***C***_*14*_	1	1	3	2	2
***C***_*15*_	0	3	3	3	0
***C***_*16*_	1	2	2	3	1
***C***_*21*_	0	3	3	3	0
***C***_*22*_	0	3	3	3	0
***C***_*23*_	1	2	1	4	1
***C***_*24*_	2	1	3	2	1
***C***_*25*_	1	1	3	2	2
***C***_*31*_	0	2	1	4	2
***C***_*32*_	1	1	3	3	1
***C***_*33*_	1	1	3	2	2
***C***_*34*_	2	1	3	2	1
***C***_*41*_	1	1	3	3	1
***C***_*42*_	1	1	3	2	2
***C***_*43*_	2	1	3	2	1
***C***_*44*_	1	1	3	3	1
***C***_*51*_	0	3	3	3	0
***C***_*52*_	1	1	3	2	2
***C***_*53*_	1	1	3	3	1
***C***_*54*_	1	1	3	2	2
***C***_*61*_	2	1	3	2	1
***C***_*62*_	0	1	4	2	2
***C***_*63*_	0	3	3	3	0
***C***_*64*_	1	2	1	4	1
***C***_*71*_	1	2	2	3	1
***C***_*72*_	1	2	1	4	1
***C***_*73*_	2	1	3	2	1

**Table 12:** C enterprise experts score statistics table.

Index	2 points	4 points	6 points	8 points	10 points
***C***_*11*_	0	3	3	3	0
***C***_*12*_	1	2	2	3	1
***C***_*13*_	0	3	3	3	0
***C***_*14*_	1	2	1	4	1
***C***_*15*_	1	1	3	2	2
***C***_*16*_	2	1	3	2	1
***C***_*21*_	1	2	2	3	1
***C***_*22*_	0	3	3	3	0
***C***_*23*_	0	1	4	2	2
***C***_*24*_	0	3	3	3	0
***C***_*25*_	1	2	1	4	1
***C***_*31*_	1	1	3	3	1
***C***_*32*_	0	1	4	2	2
***C***_*33*_	2	1	3	2	1
***C***_*34*_	1	1	3	2	2
***C***_*41*_	2	1	3	2	1
***C***_*42*_	1	1	3	3	1
***C***_*43*_	1	1	3	2	2
***C***_*44*_	2	1	3	2	1
***C***_*51*_	1	1	3	3	1
***C***_*52*_	0	3	3	3	0
***C***_*53*_	1	1	3	2	2
***C***_*54*_	0	2	1	4	2
***C***_*61*_	0	2	1	4	2
***C***_*62*_	1	1	3	3	1
***C***_*63*_	0	2	1	4	2
***C***_*64*_	2	1	3	2	1
***C***_*71*_	1	1	3	3	1
***C***_*72*_	1	1	3	3	1
***C***_*73*_	1	1	3	2	2

According to the above calculation process, it can be obtained that *B B = W*_*A*_*• R*_*A*_*=(0.1026, 0.148, 0.3089, 0.2590, 0.1217)*. By fuzzy calculation of fuzzy matrix *B* and comment set *V={2,4,6,8,10}*, the comprehensive score of *B* item can be obtained as 5.95. The project level of enterprise *B* is “standard level”; *B C = W*_*A*_*• R*_*A*_*=(0.0787, 0.1919, 0.2701, 0.2957, 0.1045)*, the fuzzy matrix *B C* and the comments set *V={2,4,6,8,10}* by fuzzy calculation, the comprehensive score of *C* project can be obtained as 5.96, and the project level of *C* enterprise is “lean level”.

**Fig. 2:**
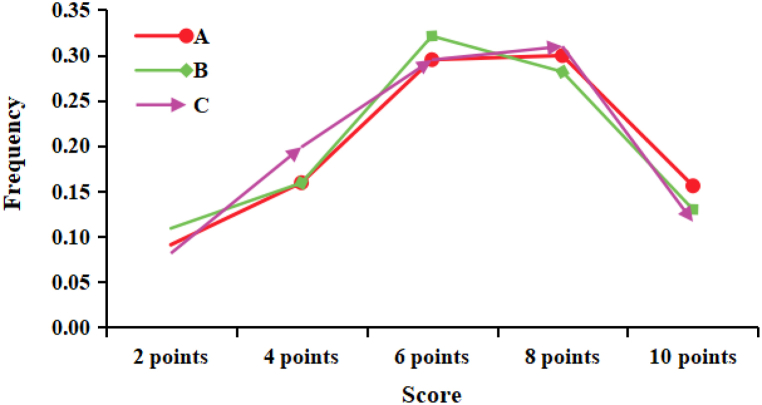
Calculation of PPP project partner selection results.

Source: This figure is drawn by the authors

Fig. 2 presents the fuzzy evaluation matrix of the three enterprises in the form of a line chart to visually compare the membership matrix of the three enterprises. *B A = W*_*A*_*• R*_*A*_*=(0.0910, 0.1594, 0.2950, 0.2996, 0.1599*). It can be seen from Fig. 2 that A has the largest probability value between *8* partitions (=0.2996), so it is finally defined as “lean level”. *B B = W*_*A*_*• R*_*A*_*=(0.1091, 0.1592, 0.3211, 0.2817, 0.1300)*, *B* has the largest probability value between *6* partitions (=*0.3211*), so it is finally defined as “standard level”; *B C = WA• RA=(0.0815, 0.1986, 0.2948, 0.3097, 0.1163)*, *C* has the largest probability value between *8* partitions (=*0.3097*), so it is finally defined as “lean level”.

According to the fuzzy evaluation matrix and the comments set *V={2,4,6,8,10}*, the final scores of *A, B and C* enterprises are *6.59*, *6.32* and *6.37* respectively. The grades of enterprise *A* and enterprise *C* are in *8* partitions (lean level), and enterprise *A*'s fuzzy calculation score of *6.59* is higher than that of enterprise *C*'s fuzzy calculation score of *6.37*.

According to the above calculation process, it can be obtained that *B B = WA• RA=(0.1091, 0.1592, 0.3211, 0.2817, 0.1300)*. By fuzzy calculation of fuzzy matrix *B* and comment set *V={2,4,6,8,10}*, the comprehensive score of item *B* can be obtained as *6.32*. The project level of enterprise *B* is “standard level”; *B C = W*_*A*_*• R*_*A*_*=(0.0815, 0.1986, 0.2948, 0.3097, 0.1163)*, the fuzzy matrix *B C* and the comments set *V={2,4,6,8,10}* by fuzzy calculation, the comprehensive score of *C* project can be obtained as *6.37*, and the project level of *C* enterprise is “lean level".

Based on the comparison of the above results, it can be seen that the comprehensive evaluation level of enterprise *A* and enterprise *C* is “lean level”, and the project score of enterprise *A* is higher than that of enterprise *C*. Therefore, according to the comprehensive evaluation results, the first company *(A)* should be selected to participate in the construction of RCEIPPPP.

### 5.4 Discussion

According to the actual situation, enterprise *A* won the bid in the *B475#* rural energy project, which is consistent with the analysis in this paper. Zhangzhuang Village is powered by 10 kV thunder *2* board, and the superior power supply is 35 kV thunder set substation. The existing public distribution of *3* units, a total capacity of 1000 kV A, electricity customers *470* households, households with 2*.*13 kV A capacity. The village has *18* rooftop photovoltaic households, with a total installed capacity of *340* kW, *9* orderly electric charging piles in the village committee square, *3 V2G* charging piles in the village cadre training school, *7* households with mechanical Wells irrigation, and *AC/DC* flexible energy storage systems are built in the village.

The data show that with the advancement of the rural energy revolution, the low-carbon and green transformation in rural areas has achieved remarkable results: the proportion of non-fossil energy consumption in the county has increased from *22* % to *75* %, the proportion of electric energy in the terminal energy consumption has increased from *37.5 %* to *64.5 %*, and the electricity generation of renewable energy in the whole society has increased from *21 %* to *94 %*. At the same time, due to the advancement of the energy revolution, the harmless treatment rate of rural household waste and livestock and poultry feces has increased from *30 %* to *100 %* and *96 %*, respectively. Through energy conservation and energy intelligence, the imbalance and inadequacy between urban and rural areas have been greatly improved. With the energy industry as the forerunner, Lankao has realized the leap-forward development of the economy and society of the whole county. The county's GDP and tax revenue grew at an average annual rate of *9.6 %* and *36.2 %*, respectively. The role of industry in promoting farmers' income is very significant: the per capita disposable income of urban residents has reached *29,900* yuan, an increase of *7.8 %*, while the per capita disposable income of rural residents has reached *16,800* yuan, an increase of *10 %*, providing more than 7000 jobs for the rural population, so that energy development and economic development are coordinated. Lankao, with its strong financial resources and strong financial resources, has effectively promoted the improvement of the economic and social development level of the county. The rural energy Internet platform with “one library and three centers” as the core is supported by the reform of rural energy production, consumption, technology, and system, so that rural energy transformation has embarked on the “fast track".

Reviewing the selection process of RCEIPPPP partners, this part uses actual cases, analyzes and calculates the data in the case through AHP and fuzzy comprehensive evaluation method, and concludes that among enterprises *A, B and C*, the comprehensive score of project *A* is 6.16, and the project grade of enterprise *A* is “lean”. The overall score of project *B* is 5.95, and the project grade of Enterprise *B* is “standard level”; The comprehensive score of project *C* is 5.96, and the project grade of enterprise *C* is “lean”. Therefore, enterprise *A* is finally selected to participate in the construction of the rural clean energy Internet project.

At the same time, there are limitations in this study, which only evaluates and makes decisions for three enterprises, and lacks many real life cases to verify the feasibility of the evaluation indicators. However, this case analysis is based on the application of scientific methods, and it is impossible to know the implementation results of the three bidding enterprises respectively. However, the construction of partner selection index in this study is the extension and modification of the existing research and the practical application of the evaluation system, which has certain scientific and practical.

The conclusions of this study are as follows: First, partners' financial ability, technical ability, management ability, performance experience, corporate reputation, cooperation ability and risk management are influencing factors for the selection of government partners for RCEIPPPPs. Compared with Ramli et al. [30], which focused on financial capability, six multi-dimensional evaluation indicators, including technical capability, management capability and performance experience, were added to implement a more rigorous and comprehensive evaluation of PPP projects. Second, compared with other factors, financial capability, technical capability, management capability and performance experience are the four key factors that are more important in selecting partners. Compared with Li [52], the direct impact of force input, technical capability, management capability and safety and environmental protection on the performance of PPP projects can be seen. The specific indicators of strength input and safety and environmental protection can be included in the indicators of performance experience and corporate reputation in this paper. On this basis, financial capability is added as a key factor in this paper. Thirdly, the empirical research shows that AHP and fuzzy comprehensive evaluation method as well as the index system constructed by this research can be applied to the practice of government partner selection for RCEIPPPPs. Compared with Zhang et al. [51], this paper is more rigorous and scientific in selecting partner selection tendency through cooperation motivation coding.

## 6 Conclusions and future prospect

### 6.1 Conclusions

This paper summarizes the dimensions and evaluation indicators of PPP partner selection for rural clean energy Internet, and uses analytic hierarchy process to empower each evaluation dimension and indicator and combines the fuzzy evaluation matrix to comprehensively evaluate the research results of the three enterprises as follows:

(1) From the perspective of PPP project operation process and the role of rural clean energy Internet partners, the selection and evaluation of partners are divided into seven dimensions: financial capability, technical capability, management capability, performance experience, corporate reputation, cooperation capability and risk management.
(2) In the evaluation of the selection of private partners for PPP infrastructure projects, the financial capacity, technical capacity and management capacity of private enterprises occupy a large proportion. In addition, due to the large weight of performance experience, in the selection of partners, in addition to the rationality of construction plans and the level of construction control concerned by traditional projects, we should also pay attention to performance experience.
(3) The comprehensive evaluation level of enterprise *A* and Enterprise *C* is “lean level”, and the project score of enterprise *A* is higher than that of enterprise C. Therefore, according to the comprehensive evaluation results, the first company (*A*) should be selected to participate in the construction of RCEIPPPP.

### 6.2 Enlightenment

The research of this paper has theoretical significance and management significance. (1) This paper constructs a partner selection system for RCEIPPPPs. This study shows that the multi-objective fuzzy comprehensive evaluation method is a powerful tool for dealing with complex partner selection problems. It can effectively take into account multiple objectives and uncertainties, providing strong support for decision-making. (2) The selection process of RCEIPPPPs is affected by many factors, including technology, economy, system and other aspects, which indicates the complexity of clean energy projects and the need for a comprehensive decision-making method. This study highlights the importance of partner selection over the life cycle of a project, and that clean energy Internet projects may need to constantly adapt and reevaluate their partners to adapt to changing market and technological conditions. (4) Managers may consider adopting multi-objective fuzzy comprehensive evaluation as a decision support tool to help them weigh different objectives and constraints in partner selection. (5) The project management team shall implement ongoing monitoring and evaluation measures to ensure that the selected partners are able to meet the project needs and objectives at different stages of the project. (6) Building a healthy partnership is essential, and project managers need to actively manage communication and cooperation with partners to ensure the successful implementation of the project.

Because the construction of the rural clean energy Internet usually lasts a long time, and needs to be oriented to the development of relatively backward rural groups, if the selected private partners occur the capital chain break, backward technology, loose management, then the progress and quality of the project can not be guaranteed [53]. Therefore, in the high-quality development of Chinese economy today, have strong financial strength, excellent technology and strong project organization and management ability, all these are the keys of whether the project can succeed or not. According to the regional characteristics of different rural areas, the government should conceive the implementation policy of clean energy Internet PPP projects, and then build a set of characteristic partner selection evaluation index system, and continue to improve it.

The index system and evaluation model of public sector partner selection under the PPP model constructed in this paper have a certain guiding role for government departments to select public sector partners. In terms of partner selection for RCEIPPPPs, the results of this study provide valuable insights for decision makers [54]. The multi-objective fuzzy comprehensive evaluation method proves to be a useful tool and can be applied to similar project selection. In addition, the project management team should recognize that project partner selection is a dynamic process that requires ongoing attention and management. Ultimately, successful project implementation depends not only on the selection of partners, but also on the establishment of good partnerships and the appropriate management measures to cope with the changing environment. These findings provide practical guidelines for the successful implementation of RCEIPPPPs.

### 6.3 Future outlook

There are certain limitations in this paper. Only three bidding enterprises of B475# rural energy engineering project are taken as realistic cases for analysis, evaluation and decision accuracy verification, and there is a lack of more relevant energy Internet engineering project cases for feasibility analysis and accuracy test of the evaluation system. With the continuous promotion of rural clean energy Internet projects in the future, The author will carry out a large number of continuous verification and improvement of the constructed evaluation system through a large number of practical cases. However, the construction of partner selection index in this study is the extension and modification of the existing research and the practical application of the evaluation system, which has certain scientific and practical. This paper designs a multi-dimensional evaluation index system for the selection of RCEIPPPP partners, provides a scientific and effective selection and decision-making method, and hopes to provide some ideas and methodological guidance for rural clean energy Internet and rural infrastructure construction [[55], [56]].

## Ethics statement

All procedures performed in this study were in accordance with the ethical standards of the university. Ethical clearance and approval were granted by Hebei Agricultural University (jgy20230505). All participants provided informed consent before joining the study.

## Funding

This research was funded by The National Social Science Fund of China grant number [22CJY043].

## Data availability statement

The data that support the findings of this study are available from the corresponding author upon reasonable request.

## CRediT authorship contribution statement

**Renjie Li:** Writing – review & editing, Software, Resources, Methodology. **Mingxuan Zhang:** Writing – review & editing, Resources, Project administration. **Shi Yin:** Writing – review & editing, Writing – original draft, Methodology. **Nan Zhang:** Writing – review & editing, Formal analysis, Data curation. **Tahir Mahmood:** Writing – review & editing, Methodology, Funding acquisition.

## Declaration of competing interest

The authors declare that they have no known competing financial interests or personal relationships that could have appeared to influence the work reported in this paper.
